# Auditory Spatial Discrimination and the Mismatch Negativity Response in Hearing-Impaired Individuals

**DOI:** 10.1371/journal.pone.0136299

**Published:** 2015-08-25

**Authors:** Yuexin Cai, Yiqing Zheng, Maojin Liang, Fei Zhao, Guangzheng Yu, Yu Liu, Yuebo Chen, Guisheng Chen

**Affiliations:** 1 Department of Otolaryngology, Sun Yat-sen Memorial Hospital, Sun Yat-sen University, Guangzhou, China; 2 Institute of Hearing and Speech-Language Science, Sun Yat-sen University, Guangzhou, China; 3 Department of Speech Language Therapy and Hearing Science, Cardiff Metropolitan University, Cardiff, Wales; 4 Department of Hearing and Speech Sciences, Xinhua College, Sun Yat-sen University, Guangzhou, China; 5 Acoustic Lab, Physics Department, South China University of Technology, Guangzhou, 510641, China; Sun Yat-sen University, CHINA

## Abstract

The aims of the present study were to investigate the ability of hearing-impaired (HI) individuals with different binaural hearing conditions to discriminate spatial auditory-sources at the midline and lateral positions, and to explore the possible central processing mechanisms by measuring the minimal audible angle (MAA) and mismatch negativity (MMN) response. To measure MAA at the left/right 0°, 45° and 90° positions, 12 normal-hearing (NH) participants and 36 patients with sensorineural hearing loss, which included 12 patients with symmetrical hearing loss (SHL) and 24 patients with asymmetrical hearing loss (AHL) [12 with unilateral hearing loss on the left (UHLL) and 12 with unilateral hearing loss on the right (UHLR)] were recruited. In addition, 128-electrode electroencephalography was used to record the MMN response in a separate group of 60 patients (20 UHLL, 20 UHLR and 20 SHL patients) and 20 NH participants. The results showed MAA thresholds of the NH participants to be significantly lower than the HI participants. Also, a significantly smaller MAA threshold was obtained at the midline position than at the lateral position in both NH and SHL groups. However, in the AHL group, MAA threshold for the 90° position on the affected side was significantly smaller than the MMA thresholds obtained at other positions. Significantly reduced amplitudes and prolonged latencies of the MMN were found in the HI groups compared to the NH group. In addition, contralateral activation was found in the UHL group for sounds emanating from the 90° position on the affected side and in the NH group. These findings suggest that the abilities of spatial discrimination at the midline and lateral positions vary significantly in different hearing conditions. A reduced MMN amplitude and prolonged latency together with bilaterally symmetrical cortical activations over the auditory hemispheres indicate possible cortical compensatory changes associated with poor behavioral spatial discrimination in individuals with HI.

## Introduction

Spatial hearing refers to the ability of the human auditory system to identify, discriminate, localize and communicate a source of sound in a complex environment [1]. It is generally accepted that spatial hearing depends on a binaural hearing system that perceives and analyzes the interaural time difference (ITD) and the interaural intensity difference (IID), together with the monaural spectral filtering of information by each pinna [2,3]. Binaural hearing plays a critical and important role in spatial hearing by enhancing sensitivity to small differences in the intensity and temporality of sound perception and consequently improves the detection and discrimination of sound sources for normal-hearing (NH) individuals [4,5].

A number of studies have indicated that the ability to detect a fine difference in binaural information is affected by sensorineural hearing loss, which causes deficit in the perception and analysis of the frequency and temporal information in sound inputs [6,7,8]. For example, Hawkins and Wiqhtman [9] found interaural time difference discrimination to be poorer in subjects with sensorineural hearing loss. Moreover, there was a significant negative correlation between the degree of hearing loss and interaural time discrimination. A suggested cause has been that there is a reduction in the number of auditory nerve fibers following hearing loss would contribute to the insensitivity to temporal fine structure, which affects the discrimination of interaural cues difference [10]

Although several studies have reported that ability of spatial hearing is affected by hearing impairment (HI), due to the poor binaural hearing effect [4,5,11,12,13,14], the benefits of binaural hearing effects appear well preserved in individuals with symmetrical hearing loss (SHL) compared to those with asymmetrical hearing loss (AHL) [5,11,12,15]. For example, Noble and Gatehouse [16] demonstrated better spatial sound-source localization and discrimination in participants with symmetrical hearing loss than those with asymmetrical hearing loss using the Speech, Spatial and Qualities of Hearing Scale (SSQ). However, another study failed to find any advantages of binaural hearing in sound localization and discrimination in individual in similar conditions using the same measurement [4]. This discrepancy in binaural hearing effect on spatial hearing may be due to subjective measurement and degree of hearing impairment.

Spatial discrimination is closely associated with extracting binaural sound information from the auditory system, which helps listeners to identify target and understand speech, particularly in difficult listening situation [17]. Spatial discrimination ability at different source positions has been well examined in NH individuals in terms of Minimum Audible Angle (MAA). MAA is defined as the angular threshold in azimuth perceived by a listener, which is an excellent tool for measuring the sensitivity of different spatial sound source locations, rather than only estimating the localization accuracy via sound source identification tasks [18,19,20]. Mills [21] was the first to report that the resolution of sound space was at least 1° at central positions, increasing to 7° at lateral positions when pure-tone stimuli were used to test NH participants.

There are however few studies that have investigated the influence of binaural hearing on spatial discrimination using the MAA in profound hearing impaired people using hearing assistant devices [8,22,23,24]. One such by Godar and Litosky [22] investigated spatial discrimination ability using MAA in children with profound HI. MAA performance was significantly improved when their second cochlear implant (CI) were activated, indicating the advantage of binaural hearing on spatial discrimination in profound hearing impaired people using hearing assistant devices.

To investigate the effect of source angle on spatial discrimination, MAA has been measured and compared in bilateral CI participants and age matched NH controls using white noise bursts at frontal and lateral position[24]. Significantly worse MAA performance was found in the lateral position in participants with bilateral CI (30°-45°) than in NH participants (7°-10°). However, no difference was found in MAA at the midline position. These results implied that hearing impaired individuals had worse spatial discrimination particularly when discriminating the sound from the lateral side due to less of the interaural difference cues [24,25].

It is noteworthy that binaural hearing effects on spatial discrimination described above were obtained from profound hearing impaired individuals with bilateral fitted CIs, rather than natural hearing impaired conditions with various audiological configurations (e.g., SHL and AHL) as well as the degree of hearing losses. The influences of these factors remain unclear.

Much evidence shows that central auditory processing is essential for accurate discrimination of sound in NH individuals [18,26]. Mismatch negativity (MMN) measurements appear to be an effective tool in detecting pre-attentive processing of spatial location changes in the central auditory system and in analyzing processing in different cortical areas [27,28,29,30]. Various studies have shown that MMN is elicited by an infrequent sound (deviant) that differs along one or more aspects from previous repetitive sound (standard), and can be used as an electrophysiological marker for the automatic process of acoustic change-detection in the auditory cortex [29] and [18,31,32]. Moreover, MMN can also be used to investigate the pre-attentive processing during passive discrimination and respective processing to left and/or right hemispheres [33].

A study by Richter et al. [33] found contralateral cortical activation responded to the spatial deviation of sounds in the ipsilateral position in NH participants. By contrast, various studies demonstrated cortical compensatory plasticity secondary to hearing loss [34,35,36,37,38,39,40,41]. Maslin et al. [42] found reduced contralateral cortical activation and subsequently increased ipsilateral activation when sound stimuli were presented to the intact ear of participants with profound unilateral hearing impairment. This implied possible cortical compensatory plasticity induced by hearing impairment due to the enhancement of synchronized neural firing in additional cortical areas.

Previous studies also suggest that cortical compensatory plasticity may be correlated with speech recognition ability [34]. Campbell and Sharma [35] compared the amplitude, latency and source localization of the N1, P2 response in individuals with mild to moderate hearing loss with NH subjects. Individuals with good speech performance exhibited a shorter P2 latency and increased activation in the temporal cortical areas when responding to nonsense speech syllables, whereas the poor performers showed increased activation over the frontal cortex. Moreover, Sandmann et al. [38] found a smaller P100 amplitude and reduced response in the visual area as well as activation of auditory cortex by visual stimulation in some CI individuals, which were negatively related to speech perception ability. These findings indicated that cortical plasticity might be one of the reasons for the reduced behavioral performance after the deprivation or degradation of auditory inputs. However, there is little knowledge as to the effect of cortical plasticity on spatial discrimination after hearing impairment.

In the present study, the aims were to investigate acoustic spatial discrimination ability at the midline and lateral positions and to explore the possible central processing mechanism in HI individuals with different binaural hearing conditions using the (MAA) and MMN responses respectively.

## Methods

### Ethics Statement

Informed consent was obtained from all participants in compliance with a protocol approved by the Institution Review Board of The Sun Yat-sen Memorial Hospital at Sun Yat-sen University of China.

### Participants

12 NH participants and 36 participants with sensorineural hearing loss (Group A), including 12 participants with SHL and 24 participants with AHL [12 with unilateral hearing loss on the left (UHLL) and 12 with unilateral hearing loss on the right (UHLR)] took part in the MAA task. A second group (Group B) was recruited for the MMN test. Group B comprised 20 NH and 60 participants with sensorineural hearing loss, which included 20 UHLL, 20 UHLR and 20 SHL participants.

Participants ranged in age from 18 to 60 years old. All were recruited for both experiments via ENT/Audiology clinics. The general information of the participant is summarized in Table 1. Hearing thresholds of the HI participants indicated mild to moderately severe sensorineural hearing loss, with no hearing threshold worse than 70 dB HL at 0.5, 1, 2 and 4 kHz in the impaired ears. Although HI participants had various hearing losses either on one side or both sides, none of them had any previous experience of wearing hearing aids before being involved in this study.

**Table 1:** General information for the participants involved in the MAA and MMN measurements.

	MAA test				MMN measurement			
	NH n = 12	UHLL n = 12	UHLR n = 12	SHLn = 12	NH n = 20	UHLL n = 20	UHLR n = 20	SHL n = 20
Male vs Female (n)	6: 6	7: 5	6: 6	7: 5	9: 11	8: 12	9: 11	10: 10
Age Mean (SD) (years)	35.58 (8.38)	37 (9.59)	34.25 (8.67)	36.17 (10.04)	35.7 (9.35)	38.25 (10.08)	36.65 (9.01)	37.0 (9.96)
Better- ear Mean (SD) (dB HL)	11.33 (3.82)	16.17 (3.79)	12.67 (4.36)	42.33 (10.09)	11.8 (3.71)	15.8 (4.47)	14.1 (5.33)	43.63 (9.83)
Worse-ear Mean (SD) (dB HL)	13.08 (4.40)	51.67 (10.42)	51.33 (9.43)	45.92 (10.23)	14.75 (4.54)	49.1 (9.65)	49.55 (9.05)	47.08 (10.08)

*Note*.MAA: minimal audible angle; MMN: mismatch negativity; NH: normal hearing; UHLL: unilateral hearing loss on left; UHLR: unilateral hearing loss on right; SHL: symmetrical hearing loss.

The inclusion criterion for the SHL group was an average between-ear difference in hearing level of less than 15 dB at 0.5, 1, 2 and 4 kHz. In contrast, the AHL group had an average between-ear difference in hearing level of greater than 15 dB [5]. Participants were excluded from the study if an air-bone gap of more than 10 dB at one or more frequencies was observed on a pure-tone audiogram. All NH and HI participants were also required to have a type A tympanogram bilaterally.

The participants involved in the Experiments MAA and MMN were matched in terms of gender, age distribution and hearing threshold of both ears. They took part in the study on a voluntary basis and no financial compensation was offered. Written consent was obtained from all participants before proceeding with any of the study procedures.

### Stimuli for the measurements

The stimuli for the measurements were white noise with a frequency ranging from 0.02 to 15 kHz. Signal duration was 1s (including 250 ms rising and falling time) and interstimulus interval was 650 ms. The stimulus generation and testing procedure were controlled using MATLAB software. The stimuli were digitally generated using a laptop computer equipped with a sound card (Terratec DMX 6Fire USB), which were subsequently attenuated/amplified and delivered via headphones (Beyerdynamic DT880 pro).

Considering space limitation, instead of free-field environment, all stimuli delivered bilaterally from different positions in the horizontal plane were enveloped by head related transfer function (HRTF) data that was obtained from the database of the South China University of Technology [43]. This method has been verified and used in various studies [44,45].

The listening level was calibrated and initially presented at approximately 65 dBA, and all participants were asked to adjust the sound to their individually comfortable listening levels at the beginning of the test. As indicated in Table 1, all HI participants had normal hearing thresholds on one side and mild to moderate hearing loss on the other side. Therefore, adequate audibility was achieved within the comfortable hearing level (normally 20–30 dB above their hearing thresholds).

### Experiment 1: experimental procedure for MAA measurement

The MAA task was conducted using a two-alternative forced choice design with a 1-up/3-down staircase paradigm. During the test, participants were required to differentiate between two standard signals (signals from the same position) and one deviant signal that differed in its angular position. The first signal was a standard stimulus and the other standard and deviant signals randomly provided within the stimulus triplets. Responses were registered by pressing buttons on the keyboard marked as option 2 or 3. The standard locations for the signals were at 0, ±45 and ±90°, with 0° tested twice using deviant signals that were presented from the right and left positions (“+” indicates the right position and “-” indicates the left position). At the beginning of each test, the first deviant stimulus had a spatial disparity of 45°. The spatial disparity between the standard and deviant sounds was reduced in the case of 3 consecutive correct responses and was increased after a false response (using an initial step size of 3° and a minimal step size of 1°). A change from a correct to a false response or a false to a correct response was marked as a turning point. A single test was ended after six turning points were reached, and the MAA threshold was set as the mean value at the last four turning points.

Participants were placed in armchairs in a sound-attenuated, low-reverberation, double-walled chamber that had been treated with acoustic-absorbing material to further reduce echoing. Prior to testing, all participants were required to perform eight practice trials. During practice trials, the correct order for the standard and deviant stimuli would be revealed after participants responded to the stimulus. The participants were asked to repeat the test after making one incorrect choice. The purpose of this session was to ensure that the participants could understand the procedure well and could correctly use the keyboard buttons.

Full instructions were given before the MAA test in order to avoid confusion between perceived changes in intensity or frequency via headphones and locational changes during the experiments. In addition, all participants were given MAA task training using various stimuli representing different intensity or frequency as well as changing the locations, in order to familiarize themselves with the MAA testing procedure, which improved the reliability and accuracy of the tests.

### Experiment 2: experimental procedure for the MMN test

The standard stimuli (probability = 0.5) was presented from the medial frontal 0° position through the headphone, whereas the deviant stimuli (probability = 0.125 each) originated from ±45° and ±90°. Each test comprised 3 blocks, with 439 mixed stimuli in each block. Forty standard stimuli were presented at the beginning of each block, with at least one standard stimulus presented before each deviant stimulus, ensuring that two contiguous deviant stimuli were different. All the auditory stimuli were presented through stimulus software E-Prime 2.0. During the test, the participants were instructed to watch a silent movie on a screen placed at the medial frontal position and to ignore the acoustic input.

The EEG signals were continuously recorded from the participants’ scalp using a 128-channel Geodesic Sensor Net and were amplified using a Net Amps 300 instrument (Electrical Geodesics Inc., Eugene, OR, USA). Electrooculogram (EOG) artifacts were monitored using four electrodes placed above and below the eyes and at the outer canthus. Prior to the EEG recording, the impedances of the individual sensors were adjusted to less than 40 kΩ [46]. All electrodes were sampled with reference to the average electrode, sample rate was 250 Hz.

After the data had been acquired, the signals were analyzed offline using Net Station software (Electrical Geodesics Inc., Eugene, OR, USA). The signals from the channels were digitally bandpass-filtered from 1 to 30 Hz. The continuous EEG was segmented into epochs starting at 200 ms before and 600 ms after the onset of the stimulus. Trials affected by eye-movements of more than 55 uV, eye blink of more than 140 uV or bad channels artefacts of more than 200 uV amplitude were rejected. After the artefact rejection, about 120–130 epochs per subject for each deviant and 660–690 standard epochs were included into subsequent analysis. Consequently, the segmented data were averaged and re-referenced to the data for the average electrode and were baseline-corrected across all tasks.

MMN response was measured and calculated by subtracting the standard response from each deviant response and determining the grand average for each group. In the present study, stimuli from different angles were used to represent the standard stimulus (at midline 0°) and deviant stimulus (at 45° or 90°) in order to reflect the laterality [47]. The latency of the MMN was defined as the negative peak in the time window of 100–300 ms, and the amplitude of the MMN was calculated within a window of ±20 ms at the peak-latency extracted from grand averaged waveform.

### Data management and statistical analyses

As part of the routine data acquisition and analysis, the MMN components were verified using a one-tailed *t*-test. However, due to all MMN amplitudes being significantly statistical different from zero, together with the complexity of the results, the verification results of the MMN responses are not presented in the manuscript.

For the statistical analysis, a repeated-measures analysis of variance (RM-ANOVA) was chosen for group comparisons as the basis for considering the potential effects of other co-variables (for example, hearing status, azimuth and direction). In the present study, the RM-ANOVA test was performed to examine the effects of hearing status (i.e., NH, UHLL, UHLR and SHL), together with azimuth and direction as nuisance covariates on MAA thresholds and MMN responses.

The mean value and standard deviation of MAA thresholds and MMN responses were calculated and compared using a post-hoc *t*-test (i.e., the Bonferroni adjustment test) when significant differences in the MAA thresholds and MMN responses were found between the groups using the RM-ANOVA. Statistical analyses were performed using the software SPSS (version 19.0, SPSS Inc., Chicago, USA). A *p*<0.05 was considered to be statistically significant.

## Results

### Demographic data and hearing thresholds for the participants

Table 1 shows general information for participants involved in Experiments 1 and 2 (i.e., MAA and MMN tests, respectively), including gender ratios, mean and standard deviation for age, and hearing thresholds of the better and worse ears.

A Chi-squared test revealed no significant difference in gender ratios (*x* ^2^ = 1.01, *p* = 0.363) between two experimental groups. Two-way ANOVA of age showed no significant main effects of hearing condition [F(1,30) = 0.007, *p* = 0.934] and group [F(1,30) = 0.302, *p* = 0.587], nor did the interaction between hearing condition and group [F(1,30) = 0.045, *p* = 0.834]. Statistical analysis for the hearing thresholds of the better [F(1,30) = 0.442, *p* = 0.551] and worse [F(1,30) = 0.043, *p* = 0.837] ears revealed no significant differences between the two experimental groups.

### Experiment 1: Comparison of MAA measurements

**Fig 1:**
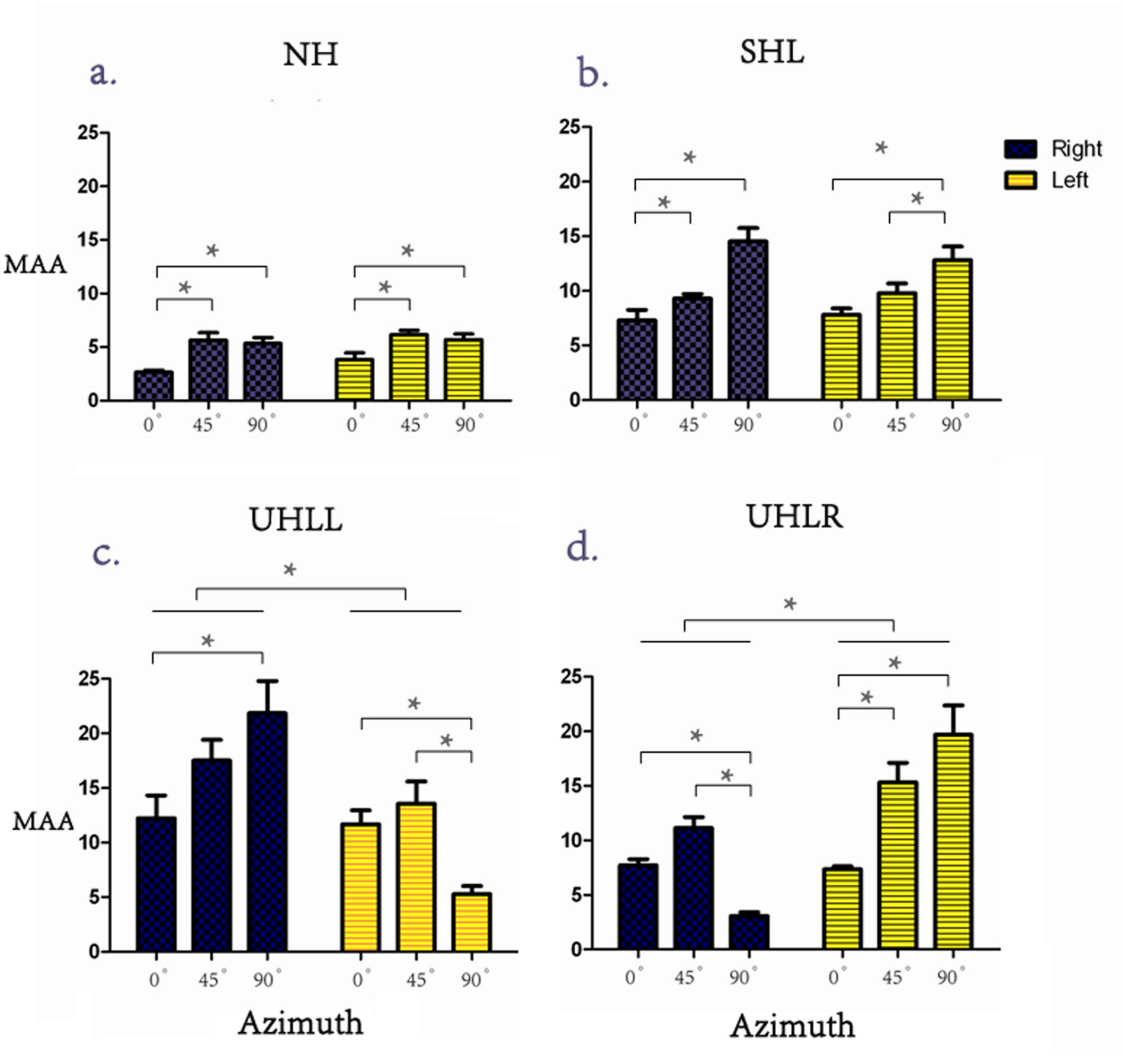
(Color Online) A comparison of the MAA performances of the subjects with all hearing abilities at the midline (0°) and lateral (45° and 90°) positions. *Note*: * there was a significant difference, i.e., *p*<0.05.

Values of MAA performance were subjected to a 4 (hearing condition: NH, SHL, UHLL, UHLR) x 3 (angle: 0°, 45°, 90°) x 2 (direction: left, right) repeated-measures ANOVA. There were significant main effects of hearing conditions [F(3,44) = 17.80, *p*<0.001] and angle [F(2,88) = 29.78, *p*<0.001], but not direction [F(1,44) = 0.014; *p* = 0.907]. Further Bonferroni-adjusted comparisons for different hearing conditions revealed that the averaged MAA was significantly smaller in the NH (4.88°) group than in the SHL (10.26°), UHLL (13.69°) and UHLR (10.73°) groups. In addition, the averaged MAA was significantly smaller in the SHL group than the UHLL group (10.26°vs. 13.69°, *p* = 0.046). A significant interaction was found among the factors of hearing condition, angle and direction [F(6,88) = 24.46, *p*<0.001]. When the interaction was further broken down by the hearing condition, follow-up analyses showed that the MAA were significantly smaller for signals originating from the 0° position than those originating from the 45° and 90° positions in the NH group [F(2,22) = 26.58, *p*<0.001] (Fig 1a). The same comparison for the SHL group showed that the MAA were significantly greater for signals originated from the 90° position than those originating from 0° and 45° positions in this group [F(2,22) = 15.57, *p*<0.001] (Fig 1b). However, direction effect was not significant in NH [F(1,11) = 2.859, *p* = 0.119] and SHL [F(1,11) = 0.151, *p* = 0.705] groups.

Participants in the UHLL group, had a significantly smaller MAA response for sound from the left side than from the right side [10.19° vs 17.19°, F(1,11) = 38.26, p<0.001]. Moreover, a significant interaction was also found between angle and direction [F(2, 22) = 20.65; *p*<0.001], which led to further analysis, revealing that the MAA threshold for the 90° position was significantly smaller than the thresholds obtained from the 0 and 45° positions when the sound was presented from the left side [F(2,33) = 9.154, *p* = 0.001]. However, a significant smaller MAA threshold was found at the 0° position than at 90° positions on the right side [F(2,33) = 4.184, *p* = 0.024] (Fig 1c).

The inverse pattern of results was obtained from the UHLR group. The MAA threshold for sounds from the right side was significantly smaller than from the left side [7.33° vs 14.13°, F(1, 11) = 17.93, *p* = 0.001]. A significant interaction was also found between the angle and direction [F(2, 22) = 20.65; *p*<0.001], and the follower-up analyses showed that the MAA threshold for the 90° position was significantly smaller than the thresholds obtained from the 0 and 45° positions when the sound was presented from the right side [F(2,33) = 35.12, *p*<0.001]. In addition, a significant smaller MAA threshold was found at the 0° position than at 45° and 90° on the left side [F(2,33) = 11.20, *p*<0.001] (Fig 1d).

### Experiment 2: Comparison of MMN amplitude and latency at electrode Fz

**Fig 2:**
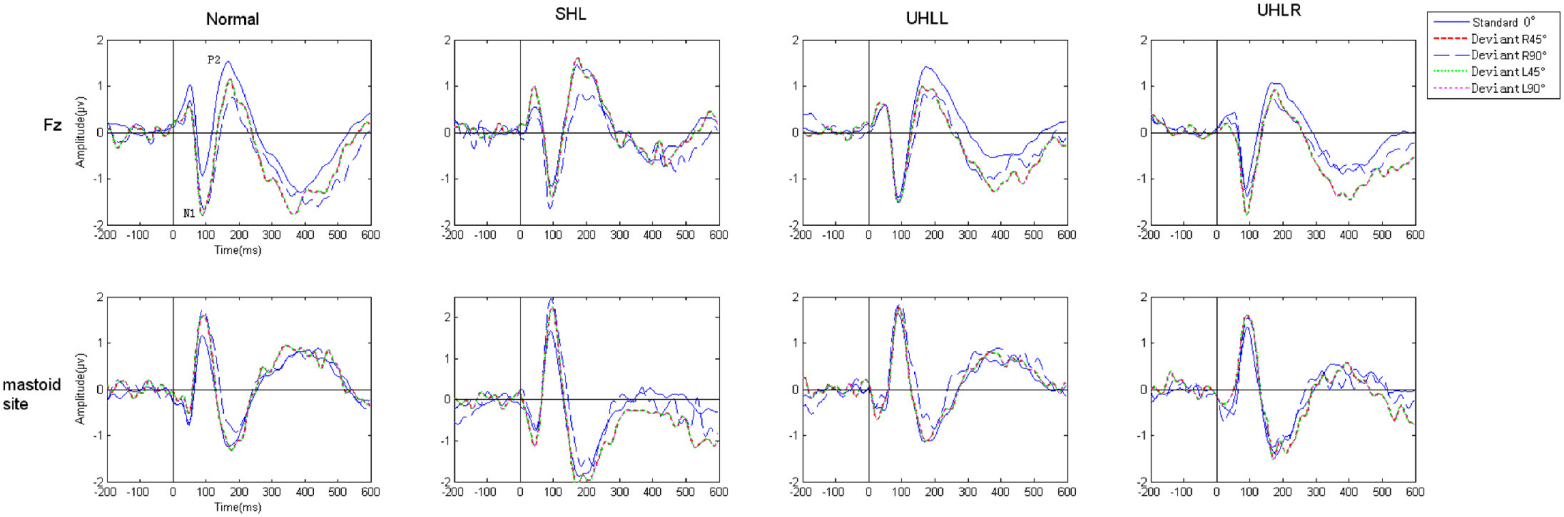
(Color Online) Response to standard and deviants at Fz and referenced mastoid site. *Note*: “R” and “L” represent right and left, respectively.

As mentioned earlier, the MMN components were verified using a one-tailed *t*-test. All MMN amplitudes showed significant statistical differences. As shown in Fig 2, the grand-average ERPs elicited by standard stimuli and deviants showed that the MMN responses could be clearly separated from the N1 wave by its longer latency, together with typically inverted polarity at mastoid sites, which implies that a deflection is not attributed to N1 refractory effect or physical stimulus differences between standard stimulus and deviants.

**Fig 3:**
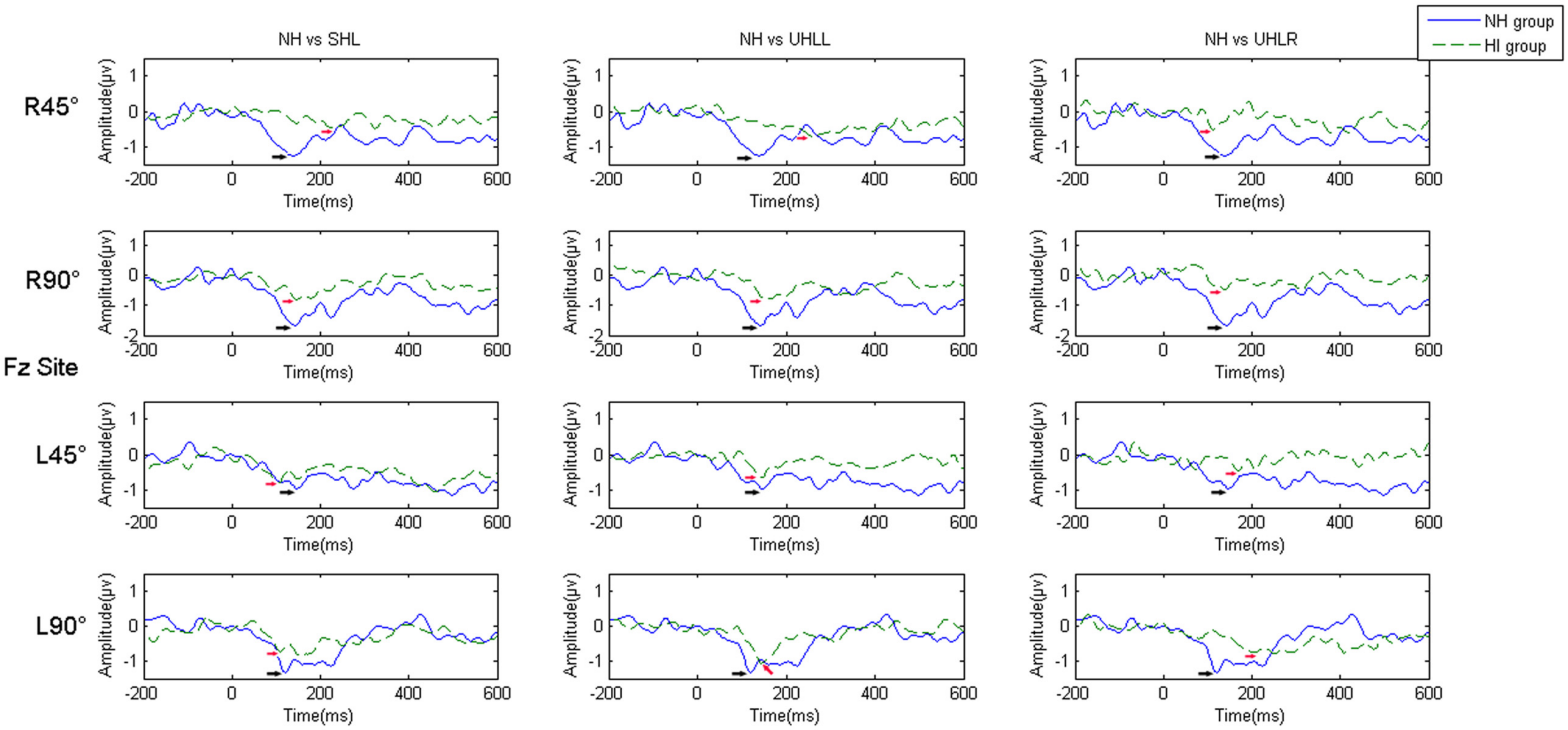
(Color Online) A comparison of the grand average MMN responses of the subjects with different hearing abilities at the Fz electrode. *Note*: the black arrow indicates the MMN response of the NH group; the red arrow indicates the MMN response of the HI group, and “R” and “L” represent right and left, respectively.

**Fig 4:**
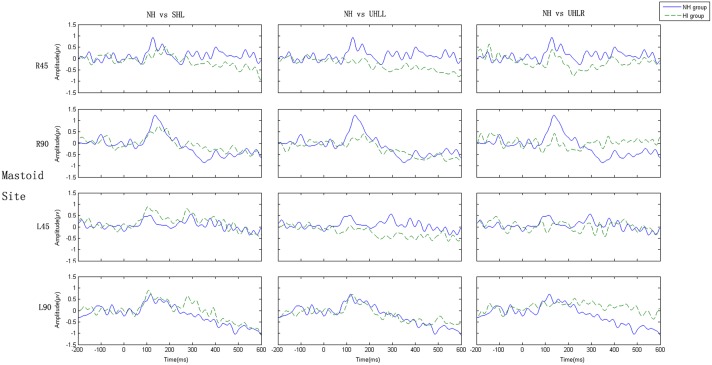
(Color Online) Grand-average difference waveforms at the referenced mastoid electrode. *Note*: “R” and “L” represent right and left, respectively.

Fig 3 presents grand average of the MMN responses elicited in participants with the four hearing conditions at the Fz electrode. Fig 4 shows the mastoid-referenced grand average difference (deviant-standard) waveforms at Fz. Values of MMN amplitude and latency were subjected to 4 (hearing condition: NH, UHLL, UHLR and SHL) x 2 (angle: 45° and 90°) x 2 (direction: left and right) repeated-measures ANOVA. In the amplitude analysis, there were significant main effects of hearing conditions [F(3,76) = 5.427, *p* = 0.002)] and angle [F(1,76) = 17.167, *p*<0.001]. Participants had larger MMN amplitudes at sound signals presented from 90° (-1.512 uV) than from 45° (-1.167uV). Further Bonferroni-adjusted comparisons for different hearing conditions revealed significantly greater amplitudes in the NH (-1.897 uV) group than those obtained from the UHLL (-1.179 uV), UHLR (-1.212 uV) and SHL (-1.068 uV) groups.

For MMN latency analysis, there was a significant effect of hearing condition [F(3,76) = 4.29, *p* = 0.008], showing that NH participants had shorter latency (182.63 ms) than participants with UHLL (211.74 ms) and UHLR (217.30 ms). However, the main effect for angle and direction did not reach significant level [F(1,76) = 0.002, *p* = 0.964; F(1,76) = 1.491, *p* = 0.227]

### Comparison of MMN amplitude at other electrodes for hemisphere effect

To investigate central processing in the left and right cortical areas, the average of the signals received by electrodes F2, F4 and Fc6 and the average of the signals received by electrodes F1, F3, and Fc5 were chosen for statistical analysis of the activity in the left hemisphere and right hemisphere, respectively. The reason for choosing these electrode sites was that the MMN responses to spatial deviant positions were primarily evoked at these frontal-central electrodes [33], which were suitable for exploring the cortical activation by spatial source stimulation. A repeated-measure ANOVA was carried out with hearing condition (NH, UHLL, UHLR, SHL), hemisphere (left, right), direction (left, right) and angle (45°, 90°) for MMN amplitude. This analysis showed significant main effects of hearing condition [F(3, 57) = 4.096, *p* = 0.012], hemisphere [F(1, 19) = 8.112, *p* = 0.013] and angle [F(1, 19) = 26.07, *p*<0.001]. A significant interaction was found between hearing and hemisphere [F(3, 57) = 2.87, *p* = 0.048], as well as between hemisphere and direction [F(1, 57) = 43.644, *p*<0.001]. In addition to explore the cortical plasticity of spatial auditory processing in individuals with different binaural hearing conditions, interaction was further broken down by hearing condition.

**Fig 5:**
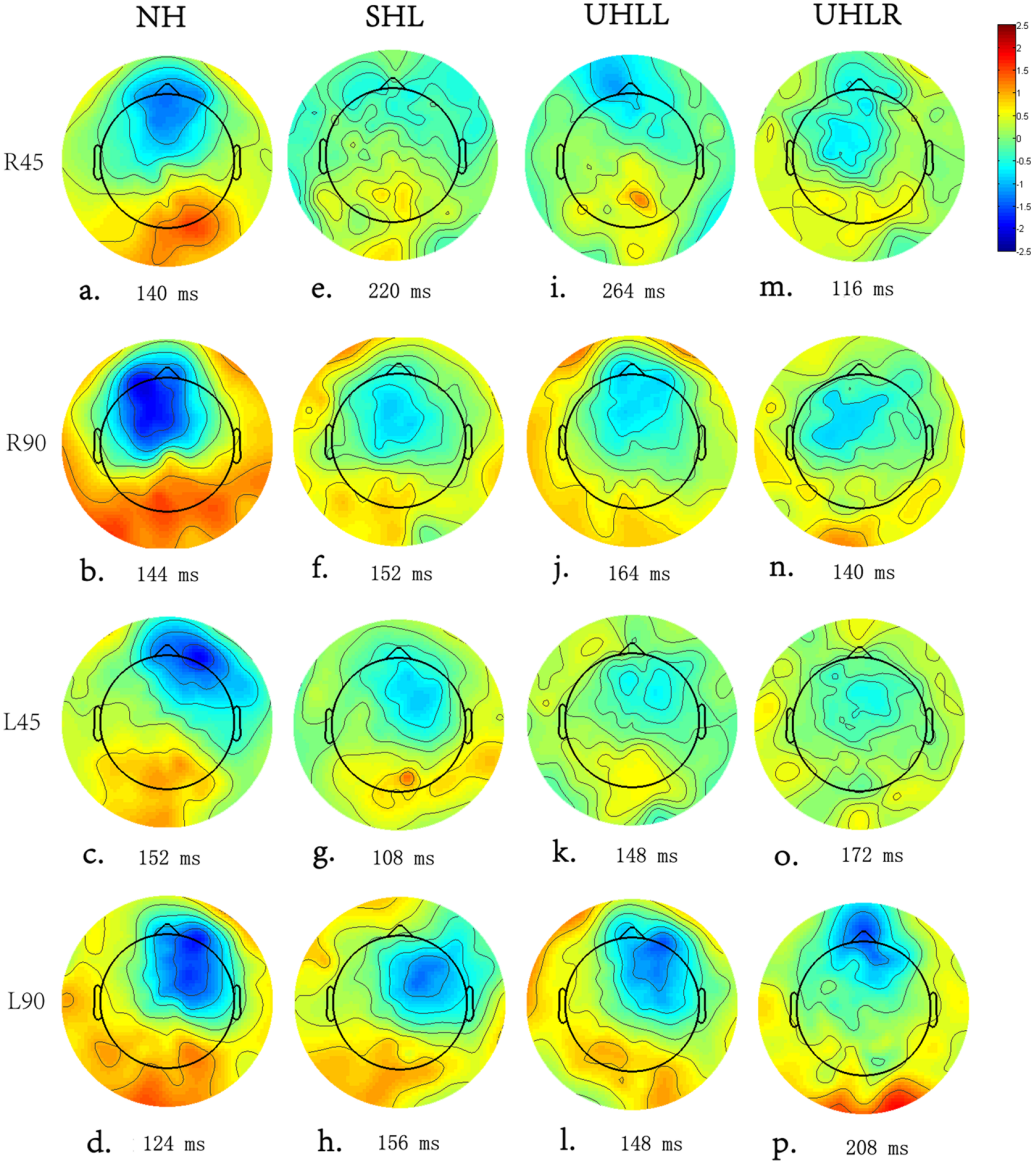
(Color Online) A topographic map based on the peak MMN responses of the subjects with different hearing abilities.

In the NH group, there was a significant interaction between hemisphere and direction [F(1, 19) = 25.85, *p*<0.001]. Follow-up analyses showed that the right hemisphere was more highly activated by a deviant sound emanating from the left side than that from the right side [F(1, 19) = 4.095, *p* = 0.05]. In contrast, a larger response over the left hemisphere was observed when the participants perceived sounds from the right side instead of the left side [F(1, 19) = 8.29, *p* = 0.011] (Fig 5a–5d).

Similarly, there was a significant interaction between hemisphere and direction [F(1,19) = 4.95, p = 0.043] for the SHL group. Further analysis showed the MMN response was significantly larger in the right hemisphere than in the left hemisphere when the SHL participants discriminated sounds emanating from the left side [F(1, 19) = 6.135, *p* = 0.027]. However, no significant difference in the MMN responses of the hemispheres was found when the sounds emanated from the right side [F(1, 19) = 0.116, *p* = 0.738] (Fig 5e–5h).

For the UHLL group (Fig 5i–5l), there were significant main effects of hemisphere [F(1, 19) = 8.15, *p* = 0.011] and significant interaction between hemisphere and direction [F(1, 19) = 4.075, *p* = 0.05]. To investigate the hemisphere effect on different direction, further RM-ANOVA was carried out with hemisphere and angle. This analysis showed significant main effects in angle [F(1, 19) = 9.57, *p* = 0.007] as well as interaction between hemisphere and angle [F(1, 19) = 7.52, *p* = 0.038] for left direction. A significantly larger MMN amplitude was found in the right hemisphere compared to the left hemisphere when UHLL participants received deviant sounds from 90° on the left direction [*t*(19) = -2.62, *p* = 0.018]. In contrast, no significant hemisphere effect was found when the sounds were presented from the right direction [F(1, 19) = 2.66, *p* = 0.121].

A significant interaction between hemisphere and direction was found for the MMN amplitude in the UHLR group [F(1, 19) = 11.59, *p* = 0.004] (Fig 5m–5p). Similar to UHLL, the RM-ANOVA (hemisphere x angle) across right and left direction showed significant hemispheric effect [F(1, 19) = 9.787, *p* = 0.007] and interaction between hemisphere and angle [F(1, 19) = 12.826, *p* = 0.003] only for right direction. Sounds originating from the 90° position on the right direction induced a significantly larger MMN response in the left hemisphere than in the right hemisphere [*t*(19) = -2.683, *p* = 0.017]. On the contrary, no significant hemisphere effect [F(1, 19) = 3.459, *p* = 0.083] or interaction [F(1, 19) = 0.092, *p* = 0.766] were found for left direction.

## Discussion

In this study, behavioral MAA and electrophysiological MMN techniques were used to examine auditory spatial discrimination and related cortical compensatory plasticity associated with conditions of hearing impairment. Overall MAA results showed that spatial discrimination was reduced with hearing impairment, although listeners with SHL had preserved the ability to spatially discriminate at a higher resolution than listeners with AHL and also exhibited a discrimination pattern similar to that of the NH subjects. In addition to the reduction in spatial discrimination an alteration in the spatial discrimination pattern was found in the listeners with AHL. Furthermore, significantly reduced MMN amplitude and prolonged latency as well as significant alteration in cortical activation was found in HI people.

### Characteristics of spatial discrimination ability in HI individuals with different binaural hearing conditions

It is generally accepted that MAA measurement is a useful tool for examining auditory spatial discrimination [18,19,20]. In subjects with normal hearing, various studies have demonstrated that auditory space discrimination is accurate at the 0° and gradually reduced when the sound comes from the side. The results obtained in Experiment 1 are consistent with the previous findings [2,3], showing better resolution spatial discrimination at the central frontal position with a decrease with increasing laterality.

In the hearing impaired (HI), although people with SHL showed a discrimination pattern similar to that of NH subjects, their auditory spatial-discrimination ability was generally reduced. This implies that the individuals with symmetrical binaural hearing made good use of binaural cues, but were less efficient due to the poor hearing sensitivity and possible frequency resolution [7].

The present study also reveals that impairment of the peripheral auditory pathway also exerts a negative effect on spatial hearing discrimination, although the processing and encoding of interaural time and intensity difference information was mainly extracted in the central auditory system [48,49]. One possible explanation for this finding is that NH individuals can accurately discriminate the sound source from the medial frontal or lateral positions because of their capacity for fine discrimination of frequency and temporal auditory information. Conversely, in HI individuals, reduction in ability to perceive subtle alterations of interaural time and intensity difference may compromise and delay spatial source discrimination. Moreover, the degraded auditory input caused by hearing impairment may further increase the cognitive load and reallocate additional cognitive resources, which consequently degrades the related spatial discrimination performance [50].

Compared to subjects with SHL, listeners with AHL exhibited greater deficits in their spatial-discrimination ability, limited degree of binaural processing and greater variability in their responses in the MAA test. It is well established that listeners with SHL outperform listeners with AHL in spatial localization and sound-source segregation in complex environments by taking advantage of their binaural hearing [4,6,11]. These findings suggest that the preservation of binaural symmetrical hearing could critically benefit the ability of auditory spatial discrimination [11].

In addition to the significant reduction in spatial-discrimination ability, an interesting finding is the alteration in spatial discrimination pattern in listeners with AHL. Listeners with AHL demonstrated better spatial resolution at 90° on the affected side than at any other position (such as the midline position). This phenomenon may be attributed to shifting the spatial position of zero IID (interaural intensity difference) from the midline in normal hearing status to a specific lateral side (for hearing impairment) [51]. Evidence has suggested that IID resolution declines with increased IID value [25]. For individual with symmetrical binaural hearing, zero IID appears at midline position, and thus the IID resolution is highest at midline. When auditory events move to the lateral positions, the IID resolution declines with increasing IID value. In contrast, for listeners with asymmetrical hearing, their zero IID shifts from midline to ipsilateral side [51], and thus the IID resolution at the ipsilateral lateral side is higher than that at midline.

### Reduced efficiency of central processing for auditory spatial discrimination in association with changes in the MMN response after hearing impairment

The results obtained from the present study showed increased MMN latency and decreased amplitude in HI individuals, consistent with previous findings [36,52]. For example, Oates et al. [52] showed an increased ERP latency in listeners with mild to moderate sensorineural hearing loss in response to the speech sounds “ba” and “da”. Moreover, Campbell and Sharma [36] demonstrated that P2 latency was increased and the P2 response more broadly distributed over the cortical areas in subjects with mild to moderate hearing loss compared with normal control subjects using speech stimuli in a passive-listening task. The latency of the ERP mainly reflects the period necessary to decode the sound stimulus in the central auditory system. Therefore, the prolongation of the MMN latency suggests that the auditory cortical system is inefficient in processing the degraded hearing inputs caused by the hearing impairment [53,54], and consequently may need to activate additional cortical regions as a compensatory cortical pathway [35]. In contrast to the MMN latency, the MMN amplitude reflects the recruitment of cortical neural resources [55,56]. The present study showed that the MMN amplitude was greatly reduced, which may be associated with a reduction in cortical-neuron recruitment and a broader distribution of neural reactivity over the auditory cortex [52]. This result supports our finding that increased MMN latency and decreased amplitude in HI individuals with longer and less efficient spatial source discrimination may contribute to the deficit in behavioral performance after hearing impairment. However, no previous studies have been conducted to explore the direct correlations between behavioral spatial discrimination performance and the related central cortical response in HI individuals. This issue needs to be addressed in future studies.

For people with NH, the MMN responses indicate that auditory stimuli can strongly activate the contralateral auditory cortex, which is supported by related neurophysiological theory on the auditory input to one ear being projected to the contralateral auditory cortex [42,57,58]. However, in the present study, the topographic distribution demonstrated cortical activation from significant contralateral cortical regions to symmetrical cortex in HI individuals, which may indicate a possible reallocation of auditory processing or a compensatory effect on cortical processing in response to auditory locational stimulation after hearing impairment. This finding is consistent with previous research in the cortical compensatory changes in people with HI [35,42]. For example, Maslin et al. [42] revealed evidence of the cortical plasticity with reduced hemispheric asymmetries after unilateral hearing impairment using scalp field topographies and source analysis of N1 AEP (auditory event potential) in a comparison of 18 unilateral hearing impaired listeners and 18 healthy listeners. Moreover, Campbell and Sharma [35] examined the N1, P2 response in individuals with mild to moderate hearing loss when responding to nonsense speech syllables. Current density reconstructions showed cortical plasticity reduced activation of the temporal cortex and increased activation of the frontal cortex, which was correlated with poor speech perception.

Indeed, established and more recent studies have advocated a form of cortical compensatory plasticity by demonstrating increased activation of the temporal auditory cortex responding to visual stimuli in association with poor speech/auditory outcomes due to hearing impairment [34,37,38,39]. For example, Lee et al. [59] found additional temporal areas recruited for visual processing in deafened people, which was evidenced by showing decreased hypometabolism in the temporal cortex when examining the HI listeners with poor speech performance using the FDG-PET technique.

Furthermore, a recent MMN study by Bottari et al. [31] demonstrated auditory cortex recruitment and reduction of activation in visual cortex when detecting visual event changes for individual with early hearing impairment. This MMN result suggests a cortical compensatory plasticity by involving the auditory cortical area in extracting and storing visual information attributed to hearing impairment.

In the present study, the finding of increased ipsilateral and reduced contralateral temporal activation in responding to lateral spatial stimuli may suggest the occurrence of possible cortical compensatory changes during discrimination of auditory spatial stimuli in parallel with increased temporal activation to visual stimuli, even in mild to moderately severe HL individual. Therefore, taken together, poor behavioral spatial discriminating outcomes, together with the evidence of decreased in MMN amplitude and prolonged latency, as well as the increased activation in bilateral symmetrical temporal areas during passive listening in various HL groups, indicates degraded behavioral spatial discrimination and possible cortical compensatory plasticity in people with HI.

Assessment of cortical compensatory plasticity due to hearing loss using either objective or behavioral measurements will become increasing relevant in the clinical setting in terms of selecting hearing-assistance strategies for HI people (e.g., bilateral hearing aids fitting or CI). In addition, it would be helpful to explore whether auditory training (e.g. auditory visual spatial localization training) or other rehabilitative procedures in conjunction with hearing aid rehabilitation may alleviate the cortical re-allocation or re-training of auditory cortices in HI individuals and consequently re-activate normal cortical neural networks during auditory processing.

A potential limitation of the present study was an inability to conduct a correlation analysis between the capabilities for spatial discrimination and the MMN responses due to the complexity and duration of the experiment, which could cause poor compliance and high drop out of participants. According to the overall aims of the present study, even though the participants for the MAA and MMN experiments were recruited from these two separate groups, the present study design and collected data were appropriate and valid because they shared similar characteristic for genders, age distribution and hearing threshold. Future research is needed to obtain evidence of a correlation between cortical compensatory plasticity and reduced spatial discrimination ability after hearing impairment. Moreover, it would be interesting to explore spatial discrimination ability of HI people in the complex environments (such as the presence of background noise).

## Conclusion

Spatial discrimination ability at midline and lateral positions vary significantly in different hearing conditions. The MAA thresholds obtained from the NH participants are significantly better than those in the HI participants. Moreover, the smallest MAA threshold was obtained at the midline position in NH participants and SHL participants However, for listeners with AHL, the best spatial resolution in terms of the MAA thresholds was found at the 90° position of the affected side. Furthermore, a reduced MMN amplitude and prolonged MMN latency together with bilaterally symmetrical cortical activation over the auditory hemispheres indicate possible cortical compensatory changes associated with poor behavioral spatial discrimination in individuals with HI.
